# Incomplete Resolution of Deep Vein Thromboses during Rivaroxaban Therapy

**DOI:** 10.1155/2017/3628127

**Published:** 2017-07-30

**Authors:** Jonathan M. Yaghoubian, Jacob Adashek, Bahareh Yaghoubian-Yazi, Menachem Nagar, Nojan Toomari, Richard J. Pietras, Uri M. Ben-Zur

**Affiliations:** ^1^College of Osteopathic Medicine of the Pacific, Western University of Health Sciences, Pomona, CA, USA; ^2^The Cardiovascular Institute of Greater Los Angeles, Tarzana, CA, USA; ^3^Faculty of Medicine, Technion-Israel Institute of Technology, Haifa, Israel; ^4^Providence Tarzana Medical Center, Tarzana, CA, USA; ^5^Division of Hematology-Oncology, Department of Medicine, David Geffen School of Medicine at UCLA, Los Angeles, CA 90095, USA

## Abstract

We present the case of a patient with a deep vein thrombosis (DVT) who failed rivaroxaban therapy. Our patient initially presented with left lower extremity edema, erythema, and pain. He was subsequently started on rivaroxaban therapy for a combined treatment period of 12 months, during and after which he persisted to have evidence of a DVT. The patient's prescribed drug regimen was changed from rivaroxaban to warfarin, which demonstrated a rapid resolution of the DVTs as determined by ultrasound assessment of our patient's lower extremity veins. Rivaroxaban, a factor Xa inhibitor, is a well-known oral anticoagulant that is used for a variety of indications and has become a mainstay in the treatment of deep vein thrombosis. With the introduction and emergence of this medication in the clinic, postmarketing reports of efficacy or lack thereof are important to review. In conclusion, we anticipate that it is likely that there are other patients with DVTs who may not respond to rivaroxaban and for whom alternative anticoagulation therapies should be explored.

## 1. Introduction

Rivaroxaban (Xarelto®) is a novel oral anticoagulant that inhibits platelet activation and fibrin clot formation via direct, selective, and reversible inhibition of factor Xa in both the intrinsic and extrinsic coagulation pathways. Factor Xa, as part of the prothrombinase complex consisting also of factor Va, calcium ions, factor II, and phospholipid, catalyzes the conversion of prothrombin to thrombin. Thrombin both activates platelets and catalyzes the conversion of fibrinogen to fibrin. At this time, factor Xa inhibitors are emerging as a popular alternative to the use of vitamin K antagonists (VKA) or warfarin therapy because factor Xa inhibitors do not require international normalized ratio (INR) testing nor specific dietary restrictions. The indications for rivaroxaban therapy include reduction in the risk of stroke and systemic embolism in patients with nonvalvular atrial fibrillation, treatment of deep vein thrombosis (DVT), treatment of pulmonary embolism (PE), reduction in the risk of recurrence of DVT and of PE after a 6-month trial for the treatment for DVT and/or PE, or prophylaxis of DVT (which may lead to PE in patients undergoing knee replacement or hip replacement surgery) [1]. Major contraindications include active pathological bleeding and severe hypersensitivity reaction such as anaphylaxis [1]. Rivaroxaban is currently widely recommended as a mainstay treatment for DVT [2]. A minimum of three months of anticoagulation with medications such as rivaroxaban are recommended in those with unprovoked DVT or recurrent DVT or provoked with minor risk factors for DVT [2]. If treatment is successful and the thrombus resolves, some patients may still require indefinite treatment [2]. Unfortunately, when factor Xa inhibitors fail, the alternatives in an outpatient setting are to change therapy to low molecular weight heparin and/or warfarin therapies [2]. Indeed, the standard for both prevention and treatment for over 60 years has been vitamin k antagonists like warfarin (Coumadin®, Jantoven®, and Marevan®), phenprocoumon, and acenocoumarol; however, VKA therapy is inconvenient due in part to its increased risk of adverse events such as bleeding, dietary restrictions, and the need for regular blood monitoring, thereby making it a less desirable option [2].

In addition to not requiring dietary restrictions nor frequent INR evaluations, rivaroxaban has other advantages such as rapid absorption and almost 100% bioavailability when taken with food (at specific doses) [3] and has been shown, in some studies, to be noninferior to enoxaparin/VKA—the current standard of care for venous thromboembolism [4]. On the other hand, rivaroxaban's disadvantages have been documented as well. The major disadvantages include internal bleeding, particularly gastrointestinal bleeding [5], as well as documented cases in which thromboembolisms do not completely resolve [6, 7].

## 2. Case Presentation

The patient in this case report is a 65-year-old white male, with a past medical history significant for mild depression and subclinical hypothyroidism, who presented in 2015 with a primary complaint of 3 days of left lower extremity pain, erythema, and edema. At the time of the visit, he also complained of dizziness of 2-day duration which was not related to changes of position. The severity of this symptom was mild to moderate, and no other associated symptoms were noted. He reported being very sedentary and experienced some tingling of the right foot. The patient denied chest pain, shortness of breath, palpitations, or syncope. He did not monitor his blood pressure at home, nor did he follow any exercise or diet programs. He has no known drug allergies, and his drug regimen at initial presentation included escitalopram 20 milligrams every other day for mild depression (which was being tapered off), as well as fiber supplements and daily multivitamins. Overall, the patient was noted to have multiple risk factors for coronary artery disease including male gender, age, sedentary lifestyle, stress, and tobacco use.

On physical examination, the patient was reported as a well-developed, well-nourished male with a BMI of 23.1 and body weight of 79.4 kilograms. The patient's neck was supple with no jugular venous distention. A left carotid bruit was audible, carotid pulses were +2/2, and there was a normal carotid artery upstroke bilaterally. His lungs were clear to auscultation bilaterally without wheezes or rhonchi. His cardiovascular exam revealed a regular heart rate and rhythm. His cardiac point of maximal impulse was at the left fifth intercostal space in the mid-clavicular line. Normal cardiac S1 and S2 sounds were noted with physiologic splitting of S2. No murmurs, thrills, rubs, gallops, or clicks were appreciated. Pulses in the extremities were 2+ throughout bilaterally at the time of presentation, and no cyanosis or clubbing was noted. Mild varicose veins in the distal lower extremities bilaterally were noted. Evidence of left lower extremity pain, erythema, and edema were noted. An abridged mental status exam revealed a cooperative patient well oriented to time, place, and person, without evidence of psychotic features. The patient's recall was within normal limits.

Given these findings, the patient underwent an ultrasound study that confirmed a left femoral vein thrombus, and he was started on rivaroxaban therapy. In accord with current recommendations for rivaroxaban therapy, the patient was started on rivaroxaban at 15 milligrams PO twice daily for 3 weeks and then continued at 20 milligrams PO daily for 6 months. Upon initiation of rivaroxaban, aspirin 81 milligrams PO daily was added to his regimen. DVT presence was monitored, and rivaroxaban therapy was eventually discontinued after a total of one year (per protocol [2]) and aspirin was continued for an additional 2 months. When serial follow-up ultrasounds continued to show persistent thrombi in the femoral and popliteal veins, he was then restarted on rivaroxaban therapy for another 2 months at 20 mg daily. After this second challenge with rivaroxaban, a repeat ultrasound again showed a persistent DVT. The patient was then bridged to warfarin therapy, continuing rivaroxaban at 20 mg daily and adding warfarin 5 mg daily with an INR goal of 2.0–3.0. Thereafter, his INR goals were met by adjusting his warfarin dose weekly. After 5 days of bridging, rivaroxaban was discontinued.

The patient was maintained on warfarin 7.5 milligrams alternating with 5 milligrams on alternate days PO (dosing varies by week per INR) in addition to escitalopram, fiber supplementation, and multivitamins, as mentioned earlier. This regimen was well tolerated by the patient and without complication.

**Figure 1:**
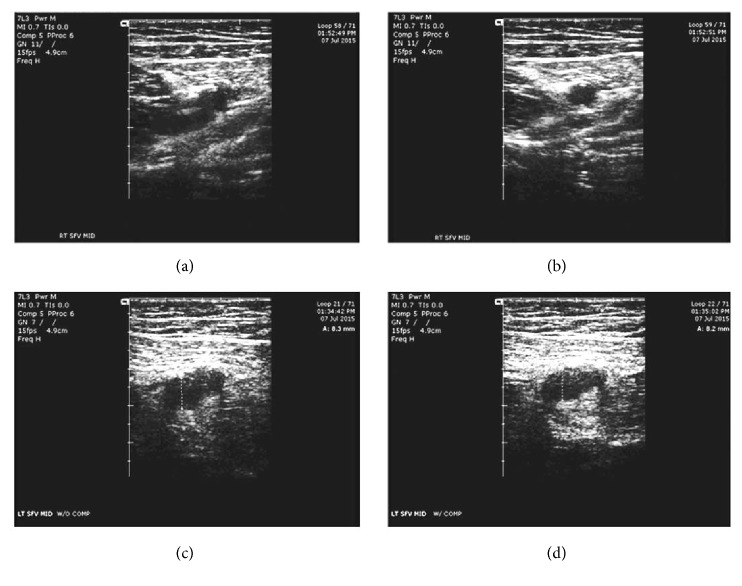
Lower extremity ultrasound findings upon initial evaluation. (a) Uncompressed right superficial femoral vein patent. (b) Compressed right superficial femoral vein completely collapsed. (c) Uncompressed left superficial femoral vein at 8.3 mm. (d) Compressed left superficial femoral vein at 8.2 mm.

**Figure 2:**
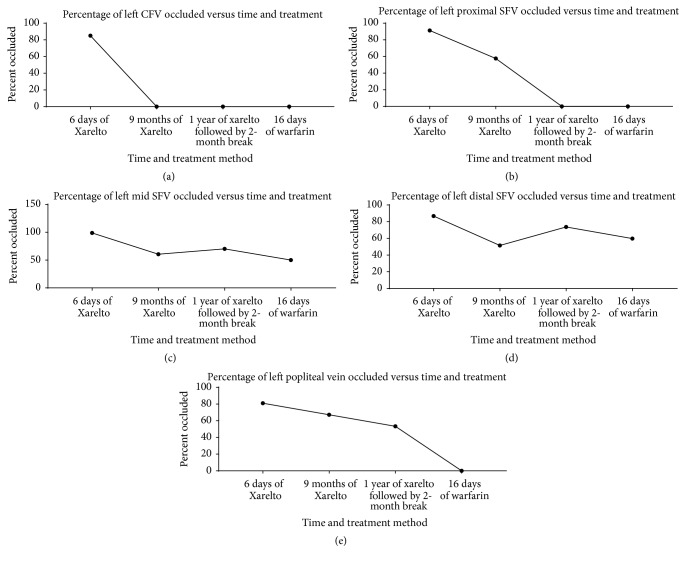
Left lower extremity vein percent occlusion. (a) Progression of vein occlusion of left CFV. (b) Progression of vein occlusion of left proximal SFV. (c) Progression of vein occlusion of left mid-SFV. (d) Progression of vein occlusion of left distal SFV. (e) Progression of vein occlusion of left popliteal vein.

In review, after initiation of rivaroxaban therapy, the patient continued to be symptomatic; therefore, we continued to monitor his DVTs to assess his progression. A Siemens ACUSON Cypress ultrasound system was used to perform ultrasonography of his lower extremity which revealed thrombus in his left mid-superficial femoral vein (SFV). The ultrasound assessments began six days after initiation of rivaroxaban therapy (Figures 1(a)–1(d)) and then again at 9 months. Then, after one year of rivaroxaban and aspirin treatment, followed by 2 months of aspirin therapy alone, another ultrasound was performed which continued to demonstrate thrombus presence. Finally, after bridging to warfarin therapy, another ultrasound was performed which demonstrated vast improvements (see Figures 2(a)–2(e)).

Figures 1(a)–1(d) demonstrated our initial/baseline ultrasound findings which correspond to six days following initiation of rivaroxaban therapy. The right mid-SFV appeared patent without compression (Figure 1(a)) and fully collapsed upon compression (Figure 1(b)), indicating no presence of thrombi. The left SFV measured 8.3 millimeters, before compression with the ultrasound transducer (Figure 1(c)), and manual compression of the vessel measured 8.2 millimeters (Figure 1(d)), indicating the presence of a thrombus.

Over a period of approximately one year of treatment with rivaroxaban, the ultrasound measurements demonstrated a general downward trend in the size of the thrombi which were followed over approximately one year of treatment with rivaroxaban, but a complete resolution in half of the vessels followed was not detected. The left common femoral vein (CFV) DVT resolved at the 9th-month mark (Figure 2(a)), and the left proximal SFV thrombosis resolved at the one-year mark (Figure 2(b)). The more distal thrombi (left mid-SFV and distal SFV) waxed and waned, but these were generally stable at the one-year assessment. When the patient was switched to warfarin treatment, there was an approximately 13% improvement in the left distal SFV and a 20% improvement of the left mid-SFV in a period of just 16 days (Figures 2(c) and 2(d)). The most drastic change in percent occlusion was of the left popliteal vein, which underwent a rapid and complete resolution of the thrombosis within 16 days of warfarin treatment after a slow but steady decline in the size of the thrombus with rivaroxaban therapy alone (Figure 2(e)).

## 3. Discussion and Conclusion

This case is of interest because a widely used and approved pharmacologic treatment of DVT did not perform as expected in this patient. A thorough literature review revealed little in the way of reports of rivaroxaban failure in the treatment of DVT, although a few case reports in which potential rivaroxaban failure is noted have emerged recently [6, 7]. Postmarketing surveillance is clearly an important factor in the further development, safety, and efficacy of any novel drug. Since the initial FDA approval of rivaroxaban in July 2011, this drug has been indicated for many uses and is widely recommended in current clinical practice [8].

Heparin and vitamin K antagonist have at times been shown to fail in the treatment of thrombi [9–11]. The recent addition of factor Xa inhibitors to anticoagulant agents is not without fault (i.e., failure, bleeding, and lack of reversal agents), as demonstrated in this case report and other papers [6, 7, 12]. The risk factors for failure of anticoagulation therapy are vast and vary from patient to patient; pharmacodynamics, pharmacokinetics, metabolism, and elimination all have a hand in how a drug interacts in a given patient and are further discussed below [13–15]. The patient discussed in our case does not seem to have any of these risk factors as noted from his normal liver function tests and creatinine clearance.

Another lab value of interest is the D-dimer level. The patient's D-dimer value was elevated to 4160 ng/ml fibrinogen equivalent units or FEU (normal is less than 500 ng/ml FEU) upon initiation of rivaroxaban treatment. The D-dimer level was noted to decrease over time with rivaroxaban alone; however, the value never normalized (even after more than one year of treatment). Over approximately 5 months of warfarin therapy alone, the D-Dimer values steadily normalized (190 ng/ml FEU).

Although factor Xa inhibitors are quite popular, there are cases emerging of its rare adverse effects (i.e., exsanguination), especially in patients with hypertension, heart failure, coronary heart disease, and impaired renal and/or hepatic function and those with certain types of cancers [16]. Animal models are exposing other mechanisms that affect the efficacy of rivaroxaban; in one mouse model, rivaroxaban's anticoagulant effect was augmented by angiotensin II [17].

The pathophysiology for the potential failure of rivaroxaban therapy in the treatment of DVT is poorly understood. However, drug metabolism may play a significant role. The physiologic metabolism of rivaroxaban is severalfold, including oxidative degradation of the morpholinone moiety as the major pathway and hydrolysis of the central amide bond and of the lactam amide bond in the morpholinone ring as the minor pathways, all of which occur within hepatocytes and liver microsomes [13]. Any change in the metabolism of any of these pathways would lead to a change in the efficacy of the drug itself. Furthermore, the main metabolite of rivaroxaban is excreted both renally and fecally via the biliary system, and malfunctioning of these pathways could also lead to a change in the efficacy of rivaroxaban. Rivaroxaban is also well known to be metabolized by the cytochrome P450 isoenzyme CYP3A4 and binds to P-glycoprotein, hence leading to risks of pharmacokinetic interactions that may alter its anticoagulant properties [14]. Furthermore, it is notable that rivaroxaban has a terminal half-life of only 5–9 hours, yet it is generally recommended to be taken at 20 mg PO once a day after an initial dosing period at 15 mg PO twice daily. In view of such relatively rapid elimination, high, early peaks of rivaroxaban could increase the risk of bleeding and low troughs could result in suboptimal anticoagulation. It will be important to determine if a better rivaroxaban dosing regimen is required to address its unique pharmacokinetic profile [15]. One study suggests a method that measures rivaroxaban's activity level in individual patients, which can be used to compare clinical variable and patient outcomes, leading to a more customizable treatment plan and dosing regimen [18].

With regard to this case report on thrombus resolution after warfarin therapy, further work-up is warranted to discover the lack of the expected response to primary rivaroxaban treatment; these diagnostics may include a thrombophilia panel and rivaroxaban serum levels to determine genetic risk factors and compliance, respectively. The patient did not present with any apparent hepatic or renal insufficiency based on normal liver function tests and creatinine levels on initial examination, as mentioned previously. Renal function has been shown to have a hand in the dosing of factor Xa inhibitors [19]. Factors that may be affecting this drug's efficacy could be lack of proper compliance, various hypercoagulable states, or certain mutations that can cause undesirable metabolism of rivaroxaban [20]. Selective serotonin reuptake inhibitors have been known to cause platelet aggregation/hypercoagulable states and potentially lead to thrombus formation and even cause pulmonary embolism [21].

Because of the threat of a pulmonary embolism from a DVT, it is important to have considered other factors/features that may be related to thrombi. A negative D-dimer lab value can help exclude the possibility of a PE [22]; in the case of our patient, initially, his D-dimer value was elevated, as noted above, and normalized after some time with warfarin therapy alone; this was not unexpected, as the protective effects of warfarin in the case of DVT/pulmonary embolism have been well documented [23].

Another feature of DVTs to consider is whether the thrombi noted in our patient are persistent or recurrent. Due to the location, initial size, and downtrending progression of the thrombi, we strongly believe that our patient had persistent thrombi. Assessing for persistent versus recurrent thrombi would be quite difficult as a thrombus can form in as little as 4 hours [24].

Although extremes of body weight (<50 kg or >120 kg) are not reported to significantly influence rivaroxaban's effects, the International Society on Thrombosis and Haemostasis (ISTH) 2016 guideline suggests avoiding the use of rivaroxaban (and other direct oral anticoagulants) in patients with a BMI >40 kg/m^2^ or body weight >120 kg due to the lack of clinical data in this population (ISTH) [25].

In clinical practice, it may be best to choose between available anticoagulant drugs on a case-by-case basis [26]. It will be important to take into account patient preferences, monitoring constraints and difficulty controlling the INR, the risk of bleeding, drug interactions, and the cost of treatment [27]. In light of this case report, we acknowledge novel oral anticoagulants, such as rivaroxaban, to be an important contribution to many formularies as they have been beneficial to the vast majority of patients who require anticoagulation; this case report is merely intended to highlight a noted anomaly to be taken into consideration for future practice.
